# Self-Humidifying Membrane for High-Performance Fuel Cells Operating at Harsh Conditions: Heterojunction of Proton and Anion Exchange Membranes Composed of Acceptor-Doped SnP_2_O_7_ Composites

**DOI:** 10.3390/membranes11100776

**Published:** 2021-10-11

**Authors:** Pilwon Heo, Mijeong Kim, Hansol Ko, Sang Yong Nam, Kihyun Kim

**Affiliations:** 1 Fuel Cell Engineering Team, Hyundai Mobis, 37, Cheoldobangmulgwan-ro, Uiwang-si 16082, Korea; pilwon@mobis.co.kr; 2 Department of Materials Engineering and Convergence Technology, Gyeongsang National University, Jinju 52828, Korea; mijeong@gnu.ac.kr (M.K.); hansol.pmc@gnu.ac.kr (H.K.); walden@gnu.ac.kr (S.Y.N.)

**Keywords:** self-humidifying membrane, acceptor doped SnP_2_O_7_, heterojunction membrane, proton exchange membrane, anion exchange membrane

## Abstract

Here we suggest a simple and novel method for the preparation of a high-performance self-humidifying fuel cell membrane operating at high temperature (>100 °C) and low humidity conditions (<30% RH). A self-humidifying membrane was effectively prepared by laminating together proton and anion exchange membranes composed of acceptor-doped SnP_2_O_7_ composites, Sn_0.9_In_0.1_H_0.1_P_2_O_7_/Sn_0.92_Sb_0.08_(OH)_0.08_P_2_O_7_. At the operating temperature of 100 °C, the electrochemical performances of the membrane electrode assembly (MEA) with this heterojunction membrane at 3.5% RH were better than or comparable to those of each MEA with only the proton or anion exchange membranes at 50% RH or higher.

## 1. Introduction

Polymer electrolyte membrane fuel cells (PEMFCs) have been intensively studied with the goal of commercialization [1,2,3]. An important issue that has recently emerged for the wide application of PEMFCs is to increase the operating temperature up to 120 °C while simultaneously decreasing the relative humidity (RH) below 30%, as suggested by the U.S. Department of Energy (DOE) [4,5]. However, commercially available perfluorosulfonic acid membranes (PFSAs), such as Nafion (Dupont Chemical, Wilgminton, DE, USA) and Aciplex (Asahi Chemical Inc., Osaka, Japan), cannot function as polymer electrolyte membranes under such harsh operating conditions because the absorbed water molecules facilitating the ion transportation are evaporated, leading to a significant decrease in the membrane conductivity [6]. To date, several promising membranes based on hydrocarbon polymers have been reported, such as polyether ether ketone (PEEK) [7], poly(arylene ether sulfone) (PAES) [8,9], polysulfone (PSf) [10], and polybenzimidazole (PBI) [11,12]. Among these membranes, the H_3_PO_4_-doped PBI membrane exhibits conductivities of ca. 10^−2^ S cm^−1^ above 100 °C and low RH condition. However, the use of a membrane electrode assembly employing the H_3_PO_4_-doped PBI membrane is problematic since some of the H_3_PO_4_ permeates into the cathode and poisons the Pt catalyst by the strong adsorption of phosphate anions [13].

As part of an alternative approach to the development of electrolyte membranes operating at high temperature and low RH condition, various composite membranes with self-humidification ability that can be prepared by simple methods have been proposed. Self-humidifying membranes are typically divided into three main types according to the materials incorporated in the membrane system: (1) Pt or Pt/C catalysts promote the reaction of hydrogen and oxygen from the anode and the cathode, respectively, to produce water in the membrane [14]; (2) hygroscopic metal oxides, such as SiO_2_ and TiO_2_, bound water molecules and maintain the hydration of the membrane [14,15]; and (3) proton-conductive materials, such as zirconium phosphate and heteropolyacids [16,17], improve the proton conducting behavior of the membrane at high temperature and low RH condition. However, although these types of membranes are useful for the harsh operating conditions of PEMFCs, they have some problems that remain unsolved: (1) the heterogeneous dispersion of Pt may lead to local electronic conduction throughout the membrane [18]; (2) the increase of the additive contents may lead to increase the ohmic resistance of the membrane [19]; (3) the poor compatibility between the additive and membrane may decrease the mechanical properties of the membrane [20].

These issues examine the heterojunction of proton and anion exchange membranes as a novel method for the development of self-humidifying membranes. When the two laminated membranes are arranged by placing the proton and anion exchange membranes at the anode and cathode sides, respectively, the following electrode reactions occur during cell discharge:

Anode: H_2_ → 2H^+^ + 2e^−^ (1)

Cathode: 1/2O_2_ + H_2_O + 2e^−^ → 2OH^−^ (2)

**Figure 1:**
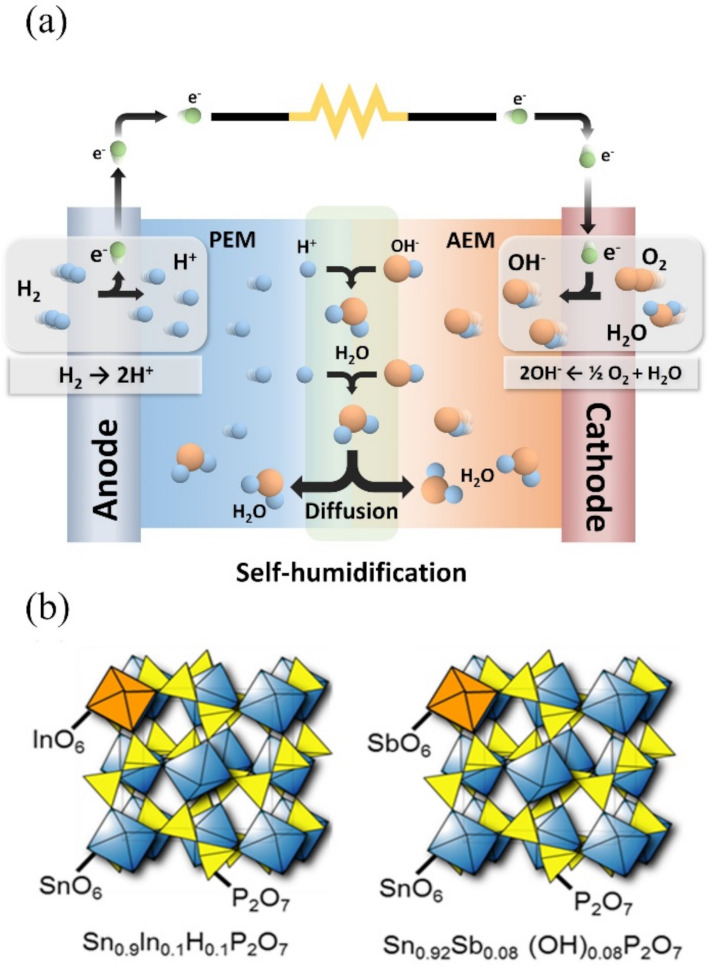
(**a**) schematic diagram of self-humidifying heterojunction membrane fuel cell and (**b**) crystalline structure of Sn_0.9_In_0.1_H_0.1_P_2_O_7_ and Sn_0.92_Sb_0.08_(OH)_0.08_P_2_O_7__._

At the interface between the two membranes, it is proposed that the following reaction occurs, as shown in Figure 1a:

2H^+^ + 2OH^−^ → 2H_2_O (3)

The resulting water product humidifies the two membranes, which improves their ionic conductivities at high temperature and low RH condition. The three problems noted above can essentially be avoided by using this technology, because it does not utilize any additives. An additional benefit of this method is the lower fabrication cost of the self-humidifying membrane, as the two membranes are simply pressed together.

In this work, acceptor-doped SnP_2_O_7_ is used as proton and anion exchange membranes because these compounds have high proton or hydroxide ion conductivity (>10^−2^ S cm^−1^) under the temperature range from 50 to 300 °C [21]. The SnP_2_O_7_ structures are characterized by the presence of intersecting zigzag tunnels delimited by pentagonal windows, which provide many ion exchangeable sites and conducting pathways [22]. The ion-exchange capabilities are incorporated into the bulk SnP_2_O_7_ through the partial substitution of Sn^4+^ cations with low valency In^3+^ and high valency Sb^5+^ due to the charge compensation of the dopant cations [18,19]. The optimal compositions of these acceptor-doped SnP_2_O_7_ compounds were determined to be Sn_0.9_In_0.1_H_0.1_P_2_O_7_ and Sn_0.92_Sb_0.08_(OH)_0.08_P_2_O_7__._ Their crystalline structures are illustrated in Figure 1b. The self-humidification of the membrane during the fuel cell operation is demonstrated by electrical conductivity and water uptake measurements. Further, the self-humidification effect on the IR loss of the membrane during the operation is quantified through electrochemical measurements.

## 2. Experimental

### 2.1. Sample Preparation

The Sn_0.9_In_0.1_H_0.1_P_2_O_7_ and Sn_0.92_Sb_0.08_(OH)_0.08_P_2_O_7_ membranes were prepared by following the previously reported procedures [20,23]: briefly, SnO_2_ and In_2_O_3_ or Sb_2_O_5_ were mixed with a H_3_PO_4_ solution at a molar ratio of P/(Sn + In or + Sb) = 2. The solution mixture was stirred at 300 °C until a high viscosity paste was formed. Next the resulting paste was calcined in a crucible alumina pot at 650 °C for 2.5 h. Then, 0.04 g of polytetrafluoroethylene (PTFE) powder was added to 1.00 g of Sn_0.9_In_0.1_H_0.1_P_2_O_7_ or Sn_0.92_Sb_0.08_(OH)_0.08_P_2_O_7_ powder, kneaded using a mortar and pestle, and finally cold-rolled to a thickness of 200 μm using a rolling mill. The two membranes obtained in this way were laminated together by pressing at 2 MPa for 2 min. For comparison, Sn_0.9_In_0.1_H_0.1_P_2_O_7_ and Sn_0.92_Sb_0.08_(OH)_0.08_P_2_O_7_ membranes with thicknesses of 400 μm were also prepared.

### 2.2. Fabrication of Hydrogen/Air Fuel Cell

Commercialized Pt/C electrode (0.5 cm^2^, Electrochem Inc., Woburn, MA, USA) with a Pt loading of 1 mg cm^−2^ was used for the preparation of the membrane electrode assembly (MEA), and the MEA was fabricated by sandwiching the electrolyte membrane between the electrodes. Two gas chambers were arranged by placing the MEA between two alumina tubes and sealing the anode side of the MEA with an inorganic adhesive. Unless otherwise stated, unhumidified hydrogen (50 mL min^−1^) and air (50 mL min^−1^) saturated by H_2_O vapor at room temperature were respectively supplied to the anode and cathode. The fuel cell was operated between 50 and 200 °C.

### 2.3. Characterization

The water uptake of the electrolyte membrane was calculated as follows. Prior to water uptake measurements, the fuel cell was operated with a current density of 80 mA cm^−2^ at 100 °C for 1 h. Then, part of the electrolyte membrane was immediately removed from the MEA and a thermogravimetric analysis (TGA; Shimadzu, DTG-60, Kyoto, Japan) was conducted from room temperature to 300 °C under air with a heating rate of 10 °C min^−1^. AC conductivity measurements of the electrolyte membrane were performed under the fuel cell operating conditions. Impedance spectra were collected using an impedance analyzer (Solartron, SI 1260, Farnborough, UK) and an electrochemical interface (Solartron, 1287, Farnborough, UK) in the frequency range from 10–10^6^ Hz with an AC amplitude of 10 mV. The IR loss of the MEA was determined using the current interruption method. During the measurement, a sharp change in voltage and a slow voltage change indicate IR loss and polarization loss, respectively. A digital oscilloscope (Nicolet, Sigma 75, Gainesville, FL, USA) was used to monitor the change in cell voltage over time.

## 3. Results and Discussion

**Figure 2:**
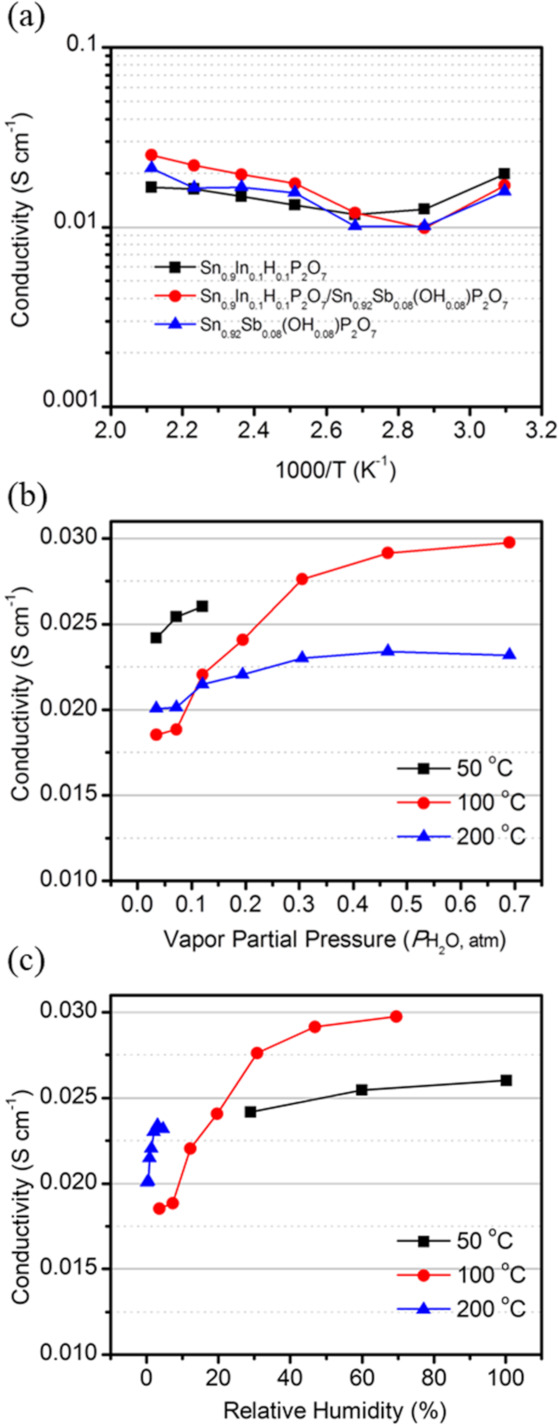
(**a**) Temperature dependency of electrical conductivity for Sn_0.9_In_0.1_H_0.1_P_2_O_7_, Sn_0.92_Sb_0.08_(OH)_0.08_P_2_O_7_, and Sn_0.9_In_0.1_H_0.1_P_2_O_7_/Sn_0.92_Sb_0.08_(OH)_0.08_P_2_O_7_ membranes. The measurement was conducted under open-circuit conditions at operating temperatures (50–200 °C) in air saturated with H_2_O vapor at room temperature. (**b**) *P*H_2_O and (**c**) RH dependency of electrical conductivity for Sn_0.9_In_0.1_H_0.1_P_2_O_7_/Sn_0.92_Sb_0.08_(OH)_0.08_P_2_O_7_ membrane.

The temperature dependency of the electrical conductivity for the Sn_0.9_In_0.1_H_0.1_P_2_O_7_/Sn_0.92_Sb_0.08_(OH)_0.08_P_2_O_7_ membrane is presented in Figure 2a. For comparison, the conductivities of the individual Sn_0.9_In_0.1_H_0.1_P_2_O_7_ and Sn_0.92_Sb_0.08_(OH)_0.08_P_2_O_7_ membranes were also included. The measurement was conducted under open-circuit conditions at the operating temperatures between 50 and 200 °C in air saturated with H_2_O vapor at room temperature. The conductivity of these membranes does not follow the Arrhenius-like dependency on temperature, because the RH decreases with increasing temperature (*P*H_2_O rather than RH was maintained constantly at the measurement). However, the electrical conductivity of the heterojunction (i.e., Sn_0.9_In_0.1_H_0.1_P_2_O_7_/Sn_0.92_Sb_0.08_(OH)_0.08_P_2_O_7_) membrane was comparable to those of the Sn_0.9_In_0.1_H_0.1_P_2_O_7_ and Sn_0.92_Sb_0.08_(OH)_0.08_P_2_O_7_ membranes within the experimental error at all tested temperatures, which implies the absence of an insulating layer at the interface of the heterojunction membrane due to the high compatibility between the Sn_0.9_In_0.1_H_0.1_P_2_O_7_ and Sn_0.92_Sb_0.08_(OH)_0.08_P_2_O_7_ membranes. A similar conclusion has been reported regarding the heterojunction electrolyte composed of proton-conductive yttrium-doped strontium zirconia and oxide ion-conductive yttria-stabilized zirconia [24].

The *P*H_2_O and RH dependency of the electrical conductivity for the Sn_0.9_In_0.1_H_0.1_P_2_O_7_/Sn_0.92_Sb_0.08_(OH)_0.08_P_2_O_7_ membrane at 50, 100, and 200 °C were also evaluated under open-circuit conditions (Figure 2b,c). Although the electrical conductivity increased with increasing *P*H_2_O at all tested temperatures, the *P*H_2_O dependency of the electrical conductivity was the largest at 100 °C. This result can be interpreted to mean the following: since the membrane is highly hygroscopic at 50 °C, high electrical conductivity is maintained even under low RH conditions. However, the evaporation of water from the membrane is significant at 200 °C, thus reducing the humidification effect on the electrical conductivity. These results suggest that the most effective temperature for the self-humidification of the fuel cell is around 100 °C. Therefore, in subsequent experiments, the fuel cell was operated at 100 °C.

**Figure 3:**
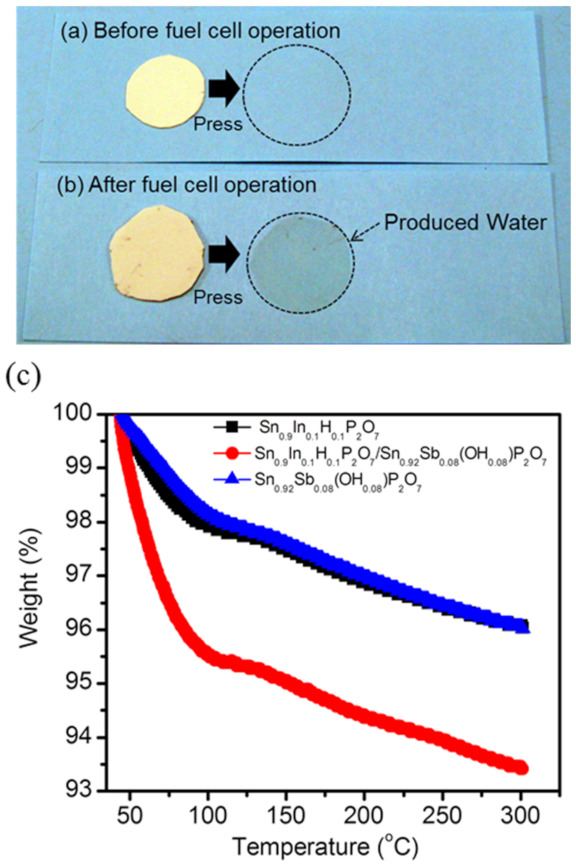
Photo images of water transfer from Sn_0.9_In_0.1_H_0.1_P_2_O_7_/Sn_0.92_Sb_0.08_(OH)_0.08_P_2_O_7_ membrane to clean paper; (**a**) before and (**b**) after fuel cell operation. Dotted circle lines indicate change of clean paper by produced water after pressing the membranes. (**c**) TGA profiles of membranes.

To observe the water product accumulated in the Sn_0.9_In_0.1_H_0.1_P_2_O_7_/Sn_0.92_Sb_0.08_(OH)_0.08_P_2_O_7_ membrane during the operation, the membranes were pressed against clean paper before and after operation (Figure 3a,b). Figure 3b clearly shows that the membrane contained a certain quantity of water after the operation that had changed compared to the quantity before the operation. Although care should be taken to account for water adsorbed on the membrane surface during cell operation, this result indicates that the observed polka-dot trace shown in Figure 3b is mainly attributable to water produced by the self-humidification effect of the membrane. Such traces are substantially less visible when using the individual Sn_0.9_In_0.1_H_0.1_P_2_O_7_ and Sn_0.92_Sb_0.08_(OH)_0.08_P_2_O_7_ membranes as electrolytes.

Further evidence of self-humidification is confirmed by a comparison of TGA profiles for Sn_0.9_In_0.1_H_0.1_P_2_O_7_/Sn_0.92_Sb_0.08_(OH)_0.08_P_2_O_7_ and the individual Sn_0.9_In_0.1_H_0.1_P_2_O_7_ or Sn_0.92_Sb_0.08_(OH)_0.08_P_2_O_7_ membranes after cell operation. Figure 3c shows that the individual Sn_0.9_In_0.1_H_0.1_P_2_O_7_ and Sn_0.92_Sb_0.08_(OH)_0.08_P_2_O_7_ membranes have similar TGA profiles: A weight loss of ca. 4% occurred in two steps between 50 and 300 °C by the desorption of physically and chemically observed water molecules, respectively [25,26]. Meanwhile the weight loss of the Sn_0.9_In_0.1_H_0.1_P_2_O_7_/Sn_0.92_Sb_0.08_(OH)_0.08_P_2_O_7_ membrane is approximately two times larger than that observed for the individual Sn_0.9_In_0.1_H_0.1_P_2_O_7_ and Sn_0.92_Sb_0.08_(OH)_0.08_P_2_O_7_ membranes. This result indicates that the increased weight loss is caused by the water produced at the heterojunction, according to Equation (3), then accumulated in the membrane.

**Figure 4:**
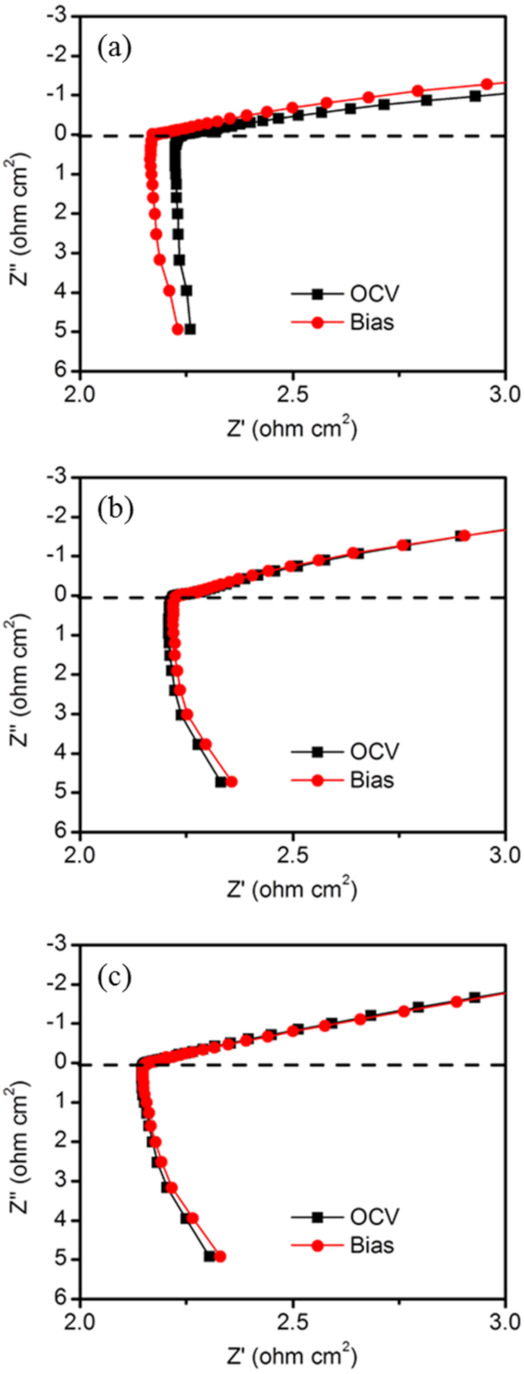
Impedance spectra for (**a**) Sn_0.9_In_0.1_H_0.1_P_2_O_7_/Sn_0.92_Sb_0.08_(OH)_0.08_P_2_O_7_ (**b**) Sn_0.9_In_0.1_H_0.1_P_2_O_7_ and (**c**) Sn_0.92_Sb_0.08_(OH)_0.08_P_2_O_7_ membranes under open-circuit and DC bias (0.3 V) conditions.

If this suggestion is correct, then a decrease in the ohmic resistance or the IR loss of the Sn_0.9_In_0.1_H_0.1_P_2_O_7_/Sn_0.92_Sb_0.08_(OH)_0.08_P_2_O_7_ membrane would be expected as a result of the operation of the fuel cell because the water produced by self-humidification increases the electrical conductivity. For clarification, electrochemical measurements were conducted. Figure 4 presents the impedance spectra of the fuel cell under open-circuit and DC bias (0.3 V) voltages conditions. The ohmic resistance of the cell with the Sn_0.9_In_0.1_H_0.1_P_2_O_7_/Sn_0.92_Sb_0.08_(OH)_0.08_P_2_O_7_ membrane was reduced by applying a DC bias voltage (Figure 4a), in contrast to the impedance spectra for the fuel cells with the individual Sn_0.9_In_0.1_H_0.1_P_2_O_7_ and Sn_0.92_Sb_0.08_(OH)_0.08_P_2_O_7_ membranes (Figure 4b,c). The ohmic resistances of the cells with the Sn_0.9_In_0.1_H_0.1_P_2_O_7_ and Sn_0.92_Sb_0.08_(OH)_0.08_P_2_O_7_ membranes remained almost unchanged, despite the fact that polarization of the fuel cells was conducted.

**Figure 5:**
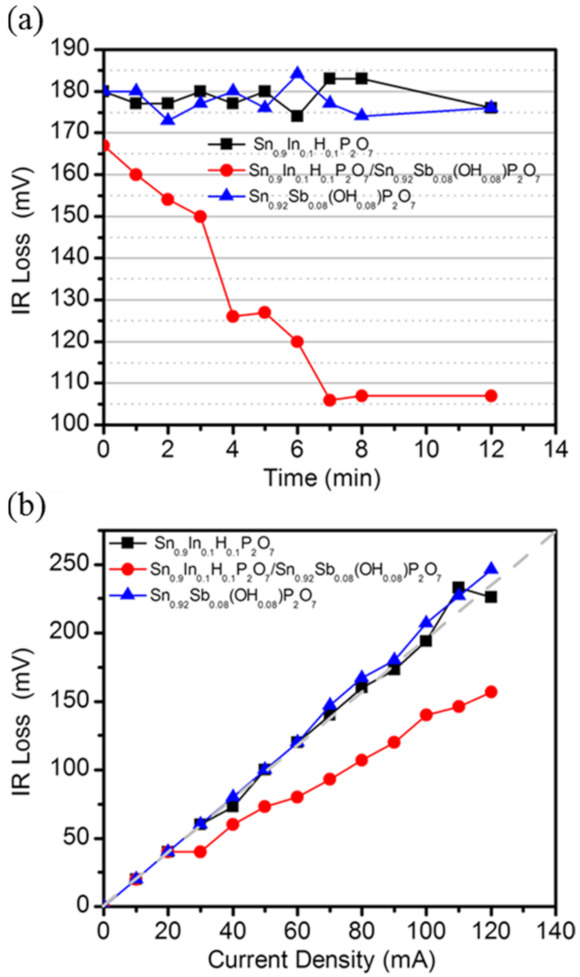
Change in IR losses for Sn_0.9_In_0.1_H_0.1_P_2_O_7_, Sn_0.92_Sb_0.08_(OH)_0.08_P_2_O_7_, and Sn_0.9_In_0.1_H_0.1_P_2_O_7_/Sn_0.92_Sb_0.08_(OH)_0.08_P_2_O_7_ membranes with respect to (**a**) time and (**b**) current density. The cells were operated at 100 °C with unhumidified H_2_ and air saturated with H_2_O vapor at room temperature (*P*H_2_O = 0.035 atm) as reactor gases for anode and cathode, respectively.

Figure 5a presents the IR losses of the fuel cell over time with a current density of 80 mA cm^−2^ applied to the fuel cells. The IR loss decreased with time until a steady state was reached. The fuel cell with the Sn_0.9_In_0.1_H_0.1_P_2_O_7_/Sn_0.92_Sb_0.08_(OH)_0.08_P_2_O_7_ membrane shows a decrement in IR loss with time, while those with the individual Sn_0.9_In_0.1_H_0.1_P_2_O_7_ and Sn_0.92_Sb_0.08_(OH)_0.08_P_2_O_7_ membranes are shown to be unchanged during the same period. These results support the self-humidification of the Sn_0.9_In_0.1_H_0.1_P_2_O_7_/Sn_0.92_Sb_0.08_(OH)_0.08_P_2_O_7_ membrane. In addition, the result of the Sn_0.9_In_0.1_H_0.1_P_2_O_7_/Sn_0.92_Sb_0.08_(OH)_0.08_P_2_O_7_ membrane also reflects an increase in water uptake with time, and the results imply that it takes approximately 7 min to reach an equilibrium of water uptake into the membrane.

Figure 5b presents the IR loss of the fuel cells as a function of the current density, where data were recorded after steady state was attained. The IR loss of the fuel cell with the Sn_0.9_In_0.1_H_0.1_P_2_O_7_/Sn_0.92_Sb_0.08_(OH)_0.08_P_2_O_7_ membrane deviated from that estimated (dotted line) from the electrical conductivity at 100 °C, the extent of which increased as the current density increased due to the enhanced self-humidification effect. By contrast, the IR losses of the fuel cells with the individual Sn_0.9_In_0.1_H_0.1_P_2_O_7_ and Sn_0.92_Sb_0.08_(OH)_0.08_P_2_O_7_ membranes almost coincided with the estimated values.

**Figure 6:**
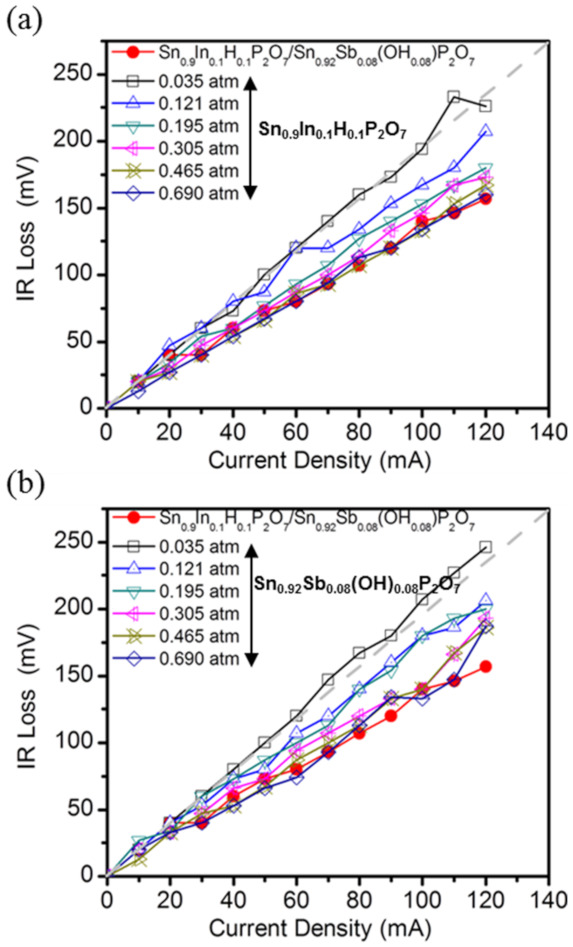
IR losses at different *P*H_2_O conditions of (**a**) Sn_0.9_In_0.1_H_0.1_P_2_O_7_ and (**b**) Sn_0.92_Sb_0.08_(OH)_0.08_P_2_O_7_ membranes as a function of current density. Red circle lines indicate IR losses of Sn_0.9_In_0.1_H_0.1_P_2_O_7_/Sn_0.92_Sb_0.08_(OH)_0.08_P_2_O_7_ membrane at a *P*H_2_O of 0.035 atm.

To clarify the self-humidification effect during the fuel cell performance, the IR losses of the fuel cells with the individual Sn_0.9_In_0.1_H_0.1_P_2_O_7_ and Sn_0.92_Sb_0.08_(OH)_0.08_P_2_O_7_ membranes with different *P*H_2_O conditions from 0.035 to 0.690 atm were measured and compared with those of the Sn_0.9_In_0.1_H_0.1_P_2_O_7_/Sn_0.92_Sb_0.08_(OH)_0.08_P_2_O_7_ membrane at a *P*H_2_O of 0.035 atm (Figure 6a,b). A comparison between the fuel cells with the Sn_0.9_In_0.1_H_0.1_P_2_O_7_/Sn_0.92_Sb_0.08_(OH)_0.08_P_2_O_7_ and individual Sn_0.9_In_0.1_H_0.1_P_2_O_7_ or Sn_0.92_Sb_0.08_(OH)_0.08_P_2_O_7_ membranes confirmed that the IR loss of the former membrane at a *P*H_2_O of 0.035 atm was equivalent to that of the latter two membranes at *P*H_2_O between 0.465 and 0.690 atm, indicating that the present heterojunction fuel cell can successfully enhance the electrolyte properties without the need for external humidification.

## 4. Conclusions

A self-humidifying fuel cell membrane showing the outstanding electrochemical performances at the harsh operating conditions of high temperature and low RH was simply prepared by laminating the proton and anion exchange membranes composed of acceptor-doped SnP_2_O_7_ composites. The effect of the heterojunction membrane (Sn_0.9_In_0.1_H_0.1_P_2_O_7_/Sn_0.92_Sb_0.08_(OH)_0.08_P_2_O_7_) on the electrolyte properties was investigated. Since the insulating layer was not formed at the interface between the Sn_0.9_In_0.1_H_0.1_P_2_O_7_ and Sn_0.92_Sb_0.08_(OH)_0.08_P_2_O_7_ membranes, the water absorption of the membrane increased during the fuel cell operation. The comparison of TGA thermograms of the membranes indicated that the water content of the heterojunction membrane was approximately two times larger than the individual Sn_0.9_In_0.1_H_0.1_P_2_O_7_ and Sn_0.92_Sb_0.08_(OH)_0.08_P_2_O_7_ membranes. Therefore, the electrochemical performances of MEAs performance with the heterojunction membrane were substantially better than those with each proton and anion exchange membranes under low RH condition. For example, the IR losses of the heterojunction membrane at current densities of 80 and 120 mA cm^−2^ after equilibrium state at 100 °C are 105 and 157 mV, respectively, while those of the Sn_0.9_In_0.1_H_0.1_P_2_O_7_ membrane are 183 and 226 mV, respectively, and those of the Sn_0.92_Sb_0.08_(OH)_0.08_P_2_O_7_ membranes are 177 and 246 mV, respectively. Based on the results, we believe that this study can provide insight into the facile preparation of a self-humidifying heterojunction membrane and the wide use of acceptor-doped SnP_2_O_7_ composites for practical application in high temperature and low RH fuel cells.
